# Biosecurity measures for the prevention of African swine fever on German pig farms: comparison of farmers’ own appraisals and external veterinary experts’ evaluations

**DOI:** 10.1186/s40813-024-00365-x

**Published:** 2024-03-11

**Authors:** Leonie Klein, Ursula Gerdes, Sandra Blome, Amely Campe, Elisabeth grosse Beilage

**Affiliations:** 1 Department of Biometry, Epidemiology and Information Processing, WHO Collaborating Centre for Research and Training for Health in the Human-Animal-Environment Interface, University for Veterinary Medicine, Hannover, Buenteweg 2, 30559 Hannover, Germany; 2 Field Station for Epidemiology, University for Veterinary Medicine, Hannover, Buescheler Strasse 9, 49456 Bakum, Germany; 3 Department of Virology, Friedrich-Loeffler-Institute, Federal Research Institute for Animal Health, Suedufer 10, 17493 Greifswald - Insel Riems, Germany; 4 Niedersaechsische Tierseuchenkasse (Animal Disease Fund), Bruehlstrasse 9, 30169 Hannover, Germany

**Keywords:** Biosecurity, Pig farms, African swine fever, Qualitative research, Biosecurity evaluation, Knowledge transfer

## Abstract

**Background:**

Since its first introduction into the German wild boar population in 2020, African swine fever (ASF) has been spreading slowly from the eastern border westwards and has been introduced into eight domestic pig farms thus far. The European Food Safety Authority has named deficits in farm biosecurity and increased human activity as major risk factors for the introduction of the ASF virus into pig farms. Studies have shown that pig farms in Germany generally have a high level of biosecurity. However, veterinary practitioners and policy-makers have expressed concerns that not all pig farmers are appropriately prepared to deal with the threat of ASF. This study aimed to evaluate the level of biosecurity on pig farms in Lower Saxony and explore the reasons for deficits in the implementation of biosecurity measures. For this purpose, pig farmers were interviewed in open structured face-to-face interviews about their perception of ASF and biosecurity, and the implemented measures on their farms were assessed with a checklist. In the data analysis, the farmers’ answers and the results of the biosecurity check were compared to gain further insights into the factors influencing the implementation of biosecurity measures on the farms.

**Results:**

The biosecurity check showed that on most farms, a high level of biosecurity had been implemented. Nevertheless, deficits were found concerning the fences and the delimitation of clean and dirty areas on farm grounds and in the anteroom. Overall, the farmers were well informed about ASF and had a realistic perception of their own biosecurity. They considered the farm layout, financial means and practicality of hygiene measures to be the main barriers to implementing biosecurity measures against ASF. However, the results also suggested that farmers’ attitudes and legal regulations were major influencing factors.

**Conclusion:**

The results indicated a high level of biosecurity against ASF on most pig farms and a realistic perception of their own biosecurity by the farmers. Current knowledge transfer and information should focus on building upon the farmers’ own motivation and expertise and supporting them to put existing knowledge into practice.

**Supplementary Information:**

The online version contains supplementary material available at 10.1186/s40813-024-00365-x.

## Background

African swine fever (ASF) is a complex viral disease that usually causes severe systemic infections and fatal haemorrhagic fever in domestic pigs and European wild boar. The virus (ASF virus, ASFV) has its roots in sub-Saharan Africa where it is transmitted in a sylvatic cycle among warthogs and soft ticks [1]. In 2007, ASFV was reintroduced to the European continent via Georgia and has now gained panzootic dimensions [2]. In Germany, ASF was first reported in 2020 [3]. The virus has been spreading slowly from the eastern border westwards within the wild boar population while also affecting domestic pig farms far away from regions with endemically infected wild boar [4–6]. To date, outbreaks on domestic pig farms have remained restricted to single farm sites (Animal Disease Notification System [7]). Nevertheless, the risk remains that ASF will be introduced repeatedly and spread further via wild boar or human activities such as pig transportation, vehicular traffic, hunting, and other routes [8].

African swine fever virus is transmitted within herds mainly via direct contact between pigs. High viral loads have been found in the blood but also in the faeces, urine and saliva of infected pigs [9]. The virus shows a high tenacity at room temperature or lower and can persist for weeks in the environment [10, 11], pork products [12] and carcasses so that inter-herd transmission most likely results from contaminated materials [13]. Via the usual oral or oro-nasal routes, relatively high doses of ASF virus are usually needed [14] for infection, but experiments have also demonstrated the infectivity of low oral doses [15, 16]. Research on the potential role of transmission via the feeding of green forage, hay and straw is ongoing [17, 18]. Over short distances, transmission might also occur via aerosols [19]. In addition, experiments have shown mechanical transmission by oral uptake of contaminated stable flies [20], but thus far, no evidence of infectious ASF virus has been found in flies in the proximity of affected pig farms [21–23]. Carrion-eating birds and rodents are discussed as mechanical vectors but are considered minor risk factors for virus transmission [24]. Epidemiological investigations into outbreaks of ASF on pig farms in Latvia, Poland and Germany have rarely been able to identify a specific entry point of the disease [13]. Deficits in farm biosecurity and increased human activity have been named risk factors in many scenarios [6, 13]. Within a pig herd, the slow spread and unspecific clinical signs of the disease increase the risk of late detection, thus also increasing the risk of further spread to other farms [25, 26]. It is generally agreed that strict biosecurity measures can prevent the introduction of ASF into domestic pig farms even in regions where ASF is endemic in wild boar [25, 27, 28]. In European legislation, ASF is listed as a disease of categories A, D, and E [29] with the aim of keeping all countries free of ASF by implementing strict preventive and, upon detection, eradication measures. The European Animal Health Law (AHL [30]) assigns responsibility for the implementation of biosecurity measures at the farm level to animal owners. It also states that animal owners should have adequate knowledge of animal diseases, biosecurity and One Health principles. Veterinarians are assigned the responsibility for raising awareness of disease prevention among animal owners. Mandatory biosecurity measures include physical and management measures [Section 1, Article 10, 4. (a), (b)]. The AHL requires all types of pig farms to implement biosecurity measures “as appropriate” and differentiates the need for further measures by disease status of the respective region. The according German law, the *Schweinehaltungshygieneverordnung* (Pig Husbandry Hygiene Ordinance) [31] indicates further detailed requirements for biosecurity measures on pig farms. It assigns different levels of mandatory biosecurity measures to pig farms according to the number of kept animals. Studies have shown that these biosecurity measures are implemented in most of the piglet-producing pig farms in Germany [32–34]. Since the reintroduction of ASF into Europe, competent authorities, agricultural associations, veterinarians and others have increased efforts to inform pig farmers about ASF as well as obligatory and advisable preventive measures. However, veterinary practitioners and policy-makers have expressed concerns that not all pig farmers are appropriately prepared to address this threat. In interviews concerning their threat perception concerning ASF, pig farmers in the German federal state of Lower Saxony, the state with the highest pig population (> 7 million pigs), said that they do not perceive the disease as an imminent threat to their livelihoods [35]. Previous studies on factors influencing farmers’ decisions concerning protective measures against animal diseases have shown that risk perception has little influence on farmers’ behaviour [36]. Studies have also shown that a lack of knowledge and a negative attitude towards biosecurity impede farmers’ implementation behaviours [37, 38], while the perceived effectiveness of biosecurity measures encourages them [39]. For these studies, farmers were usually questioned about their attitudes, perceptions, and motivations concerning measures for disease prevention. To the authors’ knowledge, however, these studies did not include an analysis of the farmers’ actual implementation behaviours.

Therefore, the aim of this study was to evaluate the level of biosecurity on pig farms in Lower Saxony and to explore possible reasons for deficits in the implementation of biosecurity measures. For this purpose, pig farmers were interviewed by the first author in open structured face-to-face interviews about their perceptions of ASF and their decisions on the appropriate biosecurity measures. Subsequently, the first author assessed the implemented biosecurity measures on these farmers’ pig farms with a checklist. In the data analysis, the farmers’ answers and the results of the biosecurity check were compared to gain further insights into the factors influencing the implementation of biosecurity measures on the farms.

## Materials and methods

### Participant recruitment

The participant group consisted of professional pig farmers from Lower Saxony selected by a two-step process. First, the farms were selected from a pseudonymized list of all pig farms that met the inclusion criteria (see Additional file 1). The list was provided by the Lower Saxony Animal Disease Fund (TSK) and comprised 9683 pig holdings, including information on the production type (farrow-to-finish, piglet producing, fattening herd) and a code. Randomly selected farmers received a letter from the Animal Disease Fund inviting them to participate in the research project. As an incentive, the farmers were offered a free biosecurity check. Interested pig farmers contacted the first author to register for the project and arrange an appointment for the interview and biosecurity check. The actual response rate decreased from 20% before to 6% during the COVID-19 pandemic, resulting in more farms randomly selected from the coded list than originally expected. Recruitment and farm visits had to be paused for a period of three months in 2020 due to restrictions caused by the COVID-19 pandemic. A total of 235 farrow-to-finish farms, 285 piglet-producing farms, and 315 fattening pig farms from the list were contacted between December 2019 and February 2021.

Outdoor pig farms were included in a separate participant group, as they face special challenges concerning biosecurity against ASF. They comprised all pig farms where pigs permanently had access to an outdoor area (e.g., a pasture or an outdoor run with concrete floor). There are few outdoor pig farms in Lower Saxony, and the coded list did not allow a specific selection by housing system. To contact a sufficient number of pig farmers with outdoor pig farms nonetheless, the local veterinary offices informed the farmers in the corresponding districts about the project. Seven outdoor pig farms were recruited directly. How many outdoor farms were contacted by the veterinary offices is unknown. All outdoor pig farmers who defined themselves as professional pig farmers (i.e., they generated a considerable amount of their income from pig production) were included in this study.

The number of participants finally included for interviews and biosecurity checks was determined by the amount of new information gained in each consecutive interview. Recruitment for further interviews stopped when theoretical saturation of information for each production type was reached [40]. According to the authors’ definition, theoretical saturation was reached when in three consecutive interviews all statements could be assigned to existing theoretical codes and no new codes were generated.

### Interview structure and procedures

Prior to the biosecurity check, the farmers were interviewed about their perception of ASF and their attitudes and decisions concerning biosecurity measures on their farms (see also [35]). The open, structured interview guide (see Additional file 2) was designed by the first author and reviewed and discussed by the co-authors and three veterinarians specialised in pig farming. It was adjusted according to their feedback, and test interviews were conducted on five pig farms that were then excluded as participants in the final project. All interviews were conducted in German by the first author on the participants’ farms. Interviews were audio recorded and later transcribed by the first author using the software f4transcript (Dr. Dresing & Pehl GmbH, Deutschhausstrasse 22a, 35037 Marburg, Germany, www.audiotranskription.de). Separate notes taken during or immediately after the interviews included information about the interview situation and provided further input for later analysis.

### Interview analysis

Interview transcripts were analysed by the first author with qualitative content analysis [41, 42]. For the interview analysis, the software f4analyse [43] was used. The initial code system was developed deductively from the first eight interviews based on the interview questions. Additional themes and subcategories were added in an inductive approach during the analysis process (for more details, see [35]).

In addition, the quantitative results from the content analysis were exported into Microsoft^®^ Excel^®^2016 (Version 16.0.5356.1000) and analysed by applying descriptive data analysis in SAS© (Version 9.4 [44]) to determine how many pig farmers had responded in similar ways and which themes were mentioned most often in the interviews. It is important to keep in mind, however, that the interviews only included open interview questions, and therefore, it is not possible to draw decisive conclusions about missing mentions of certain topics [45].

### Evaluation of farm biosecurity

For the evaluation of the biosecurity measures implemented on the farms, a checklist was developed based on the German Pig Husbandry Hygiene Ordinance (*Schweinehaltungshygieneverordnung* [31]), various checklists that veterinary authorities use during their control visits and an online tool developed for farmers to evaluate their own biosecurity measures against ASF, the *ASF-Risikoampel* (translated as traffic light for ASF risk, online available at https://risikoampel.uni-vechta.de/). Prior to the development of the checklist, risks for the introduction of ASFV into pig farms were discussed among the authors and with veterinarians from three veterinary offices in Lower Saxony. As a result of their feedback, the checklist was condensed to focus on those biosecurity measures that were considered most important to prevent the introduction of ASFV into pig farms. The initial draft of the checklist was then reviewed and discussed with the co-authors and with five veterinary practitioners who specialised in pigs and refined according to their feedback. In a pilot phase, the checklist was tested on five pig farms that did not participate in the final project. The final biosecurity checklist consisted of 62 questions (see Additional file 3).

The pigs were often situated at more than one farm site location, and biosecurity evaluations were performed at as many farm sites as the pig farmers wanted and time allowed. In the biosecurity check, farm sites were differentiated by location, layout, and ownership. The main farm sites were the farmers’ primary production sites, usually situated in residential areas, comprised of multiple buildings including the farmers’ homes. Leased farm sites were similar to the main production sites in location and layout but were leased and not owned by the interviewed farmers. Farm sites next to the main site or separately located farm sites were usually located outside of residential areas and consisted of only one pig barn. Outdoor farm sites were defined as all sites where pigs were kept in pastures or pig barns with an outdoor area. All farm sites, irrespective of size and production type, were evaluated with the same checklist and by the same criteria. During farm visits, the checklists were replenished with survey maps of the farm sites to support the later analysis of the data.

### Analysis of biosecurity evaluation

The data from the biosecurity checklists were coded in Microsoft^®^ Excel^®^2016 (Version 16.0.5356.1000) and analysed by applying descriptive statistical analysis in SAS© (Version 9.4).

To condense the results, enable a comparison between production types and simplify the joint analysis of the biosecurity evaluation and interview statements, a scoring system was developed (see Additional file 4). The scoring system assigned the questions from the checklist to nine biosecurity subsets: (1) perimeter fences, (2) building structure of pig barns and pastures, (3) pathways on farm grounds and vehicle hygiene, (4) loading areas, (5) construction and fencing of feed silos, (6) storage of feed and bedding materials, (7) shoe hygiene measures, (8) anteroom facilities, and (9) rodent control measures. Measures in these subsets were rated from zero (low biosecurity) to two (high biosecurity) depending on how far biosecurity measures had been implemented. The scoring system was based on considerations about the highest risks for the introduction of ASF into pig farms, as discussed in scientific literature and reports from affected regions, as well as assessments from veterinary epidemiologists. The range and distribution of scores were then also analysed by applying descriptive statistical analysis in SAS© (Version 9.4).

### Joint analysis of biosecurity evaluation and comments by farmers

For the joint analysis, statements from the interviews, in which farmers evaluated the biosecurity measures at their farm sites, were coded and scored similarly to the checklists. A score of 0 meant that, in the farmer’s opinion, a biosecurity measure was missing or not sufficiently implemented; a score of 1 indicated that the farmer considered the measure to be implemented with room for improvement; and a score of 2 indicated that the farmer regarded the biosecurity measure as fully and correctly implemented. Farmers were not specifically asked to rate specific biosecurity measures in the interviews, but rather their evaluation of their own biosecurity measures was the result of more general questions about how they perceived their own biosecurity measures as prevention of an introduction of ASFV and where they saw deficits in their own biosecurity measures. When farmers held pigs at more than one farm site, general comments about the implementation of specific biosecurity measures on the farm were coded accordingly for all farm sites. If farmers specified the farm site in the comment, the score was only given to that farm site. The scores given by the farmers were then compared to the scores from the checklist by applying descriptive statistical analysis in SAS© (Version 9.4). The statistical unit for the analysis was the farm site.

## Results

### Participants

In total, 81 pig farmers were recruited for participation: 28 with farrow-to-finish (ftf), 17 with piglet-producing (pi), 22 with fattening pig (fa) and 14 with outdoor pig farms (of). Among pig farms with indoor housing systems (ftf, pi, fa), the farms ranged in size from 80 to 1200 sows (pi and ftf, median: 300) and from 205 to 6000 fattening pigs (fa, median: 2400), and among outdoor pig farms from 50 to 1500 fattening pigs (median: 50) and from 4 to 850 sows (median: 10). Approximately half of the farms with indoor housing systems produced in a multi-site system, while most of the outdoor farms had a one-site system (Table 1). In total, biosecurity measures were evaluated at 47 farrow-to-finish, 27 piglet-producing, 38 fattening pig and 16 outdoor pig farm sites.

**Table 1:** Number of production sites of participating pig farms

Production type	Amount of farm sites				All
	1	2	3	4	
Farrow-to-finish farms	13	13	0	2	28
Piglet-producing Farms	11	2	4	0	17
Fattening pig farms	9	10	3	0	22
Outdoor farms	13	1	0	0	14

### Farmers’ evaluations of their biosecurity measures and results from the biosecurity check

In total, 588 evaluations of specific biosecurity measures were coded in the interviews, varying from two to ten evaluations per interview (median: ftf: 5, pi: 5, fa: 4.5, of: 4.5). Farmers spoke more often and in more detail about biosecurity subsets that they considered critical on their farm sites than about areas of less importance for them (Table 6). More than half of their evaluations concerned perimeter fences, anterooms and shoe hygiene measures.

### Perimeter fences

Most of the farmers evaluated the implementation of a perimeter fence as one of the most important biosecurity measures for ASF prevention (Table 6). Not only is a fence obligatory for pig farms of a certain size [31], but farmers also perceive it as effective against the introduction of animal diseases.

“Well, against ASF, the most important measure is the protection to the outside, right? Since ASF is here, we have built an additional fence, and if it comes closer, you have to watch slightly more intensively that it is closed” (Interview-nr. 40, ftf)

Nevertheless, among farmers of piglet-producing and fattening pig farms, more than one-third perceived their fences as inadequate (score 2: ftf: 44.7% n = 21, pi: 22.2% n = 6, fa: 21.1% n = 8, of: 43.8% n = 7; score 0: ftf: 12.8% n = 6, pi: 37.0% n = 10, fa: 44.7% n = 17, of: 0%). According to the results of the biosecurity check, at least one-third of farm sites were not sufficiently fenced, most among fattening and outdoor pig farms (score = 0, for criteria see the Scoring System in the supplementary materials) (Table 2, 3, 4, 5). The implementation or lack of fences in multisite systems was often consistent within one farm (and therefore for one farm manager). For seven out of eight outdoor farmers, the biosecurity check indicated that the farms were less securely fenced than the farmers claimed. (Table 6). These farmers considered the double fence around the pig pastures to be sufficient protection and saw no need for an additional perimeter fence around the whole farm site. Major obstacles to the construction of fences among all pig farmers were the high costs and impracticality of fencing farm sites consisting of multiple buildings. These obstacles outweigh the relatively low perceived risk of being directly affected by ASF in the near future.

“However, as I said, we are located in the village and the veterinarian has been here before; she also said then, "Yes, if the wild boars run over here from the federal highway eventually…" However, that is something I cannot imagine, that wild boars truly run on the road here” (Interview-nr. 64, ftf).

**Table 2:** Implementation of biosecurity measures at different farm sites of farrow-to-finish farms

Biosecurity subset	Score	At main site		Next to main site		At separate site		At leased farm site		Outdoor farm site		All	
		n	%	n	%	n	%	n	%	n	%	n	%
*Farrow-to-finish farms (n = 47)*													
Perimeter fence	0	10	38.46	2	66.67	4	33.33	4	80.00	0	0	20	42.55
	1	3	11.54	0	0	3	25.00	0	0	0	0	6	12.77
	2	13	50.00	1	33.33	5	41.67	1	20.00	1	100.00	21	44.68
	− 99	0	0	0	0	0	0	0	0	0	0	0	0
Building structure	0	0	0	0	0	0	0	0	0	0	0	0	0
	1	9	34.62	0	0	1	8.33	1	20.00	1	100.00	0	0
	2	17	65.38	3	100.00	11	91.67	4	80.00	0	0	12	25.53
	− 99	0	0	0	0	0	0	0	0	0	0	35	74.47
Pathways/vehicle hygiene	0	5	19.23	0	0	1	8.33	3	60.00	1	100.00	10	21.28
	1	5	19.23	2	66.67	6	50.00	1	20.00	0	0	14	29.79
	2	15	57.69	1	33.33	5	41.67	1	20.00	0	0	22	46.81
	− 99	1	3.85	0	0	0	0	0	0	0	0	1	2.13
Loading ramps	0	6	23.08	0	0	1	8.33	2	40.00	1	100.00	10	21.28
	1	4	15.38	0	0	1	8.33	2	40.00	0	0	7	14.89
	2	15	57.69	3	100.00	8	66.67	1	20.00	0	0	27	57.45
	− 99	1	3.85	0	0	2	16.67	0	0	0	0	3	6.38
Feed silos	0	6	23.08	2	66.67	2	16.67	2	40.00	0	0	12	25.53
	1	16	61.54	1	33.33	8	66.67	2	40.00	0	0	27	57.45
	2	4	15.38	0	0	2	16.67	1	20.00	1	100.00	8	17.02
	− 99	0	0	0	0	0	0	0	0	0	0	0	0
Enrichment materials	0	1	3.85	1	33.33	0	0	0	0	0	0	2	4.26
	1	0	0	0	0	0	0	0	0	0	0	0	0
	2	25	96.15	2	66.67	12	100.00	5	100.00	1	100.00	45	95.74
	− 99	0	0	0	0	0	0	0	0	0	0	0	0
Shoe hygiene	0	8	30.77	0	0	2	16.67	1	20.00	1	100.00	12	25.53
	1	7	26.92	0	0	2	16.67	1	20.00	0	0	10	21.28
	2	11	42.31	3	100.00	8	66.67	3	60.00	0	0	25	53.19
	− 99	0	0	0	0	0	0	0	0	0	0	0	0
Anteroom	0	0	0	0	0	1	8.33	0	0	0	0	1	2.13
	1	8	30.77	0	0	2	16.67	4	80.00	0	0	14	29.79
	2	18	69.23	3	100.00	9	75.00	1	20.00	1	100.00	32	68.09
	− 99	0	0	0	0	0	0	0	0	0	0	0	0
Rodent control	0	1	3.85	0	0	1	8.33	1	20.00	0	0	3	6.38
	1	5	19.23	0	0	4	33.33	0	0	0	0	9	19.15
	2	20	76.92	3	100.00	6	50.00	4	80.00	1	100.00	34	72.34
	− 99	0	0	0	0	1	8.33	0	0	0	0	1	2.13

Scores: 0 = “no biosecurity measures have been implemented, low biosecurity”, 1 = “some biosecurity measures have been implemented, moderate biosecurity”, 2 = “all necessary biosecurity measures have been implemented, high biosecurity”, − 88 = not applicable, − 99 = missing data

**Table 3:** Implementation of biosecurity measures at different farm sites of piglet-producing farms

Biosecurity subset	Score	At main site		At separate site		At leased farm site		All	
		n	%	n	%	n	%	n	%
*Piglet-producing farms (n = 27)*									
Perimeter fence	0	7	58.33	5	50.00	3	60.00	15	55.56
	1	2	16.67	0	0	0	0	2	7.41
	2	3	25.00	5	50.00	2	40.00	10	37.04
	− 99	0	0	0	0	0	0	0	0
Building structure	0	0	0	0	0	0	0	0	0
	1	4	33.33	0	0	0	0	4	14.81
	2	8	66.67	10	100.00	5	100.00	23	85.19
	− 99	0	0	0	0	0	0	0	0
Pathways/vehicle hygiene	0	3	25.00	0	0	1	20.00	4	14.81
	1	4	33.33	8	80.00	3	60.00	15	55.56
	2	5	41.67	2	20.00	1	20.00	8	29.63
	− 99	0	0	0	0	0	0	0	0
Loading ramps	0	4	33.33	1	10.00	2	40.00	7	25.93
	1	0	0	0	0	0	0	0	0
	2	7	58.33	9	90.00	3	60.00	19	70.37
	− 99	1	8.33	0	0	0	0	1	3.70
Feed silos	0	2	16.67	0	0	2	40.00	4	14.81
	1	9	75.00	5	50.00	3	60.00	17	62.96
	2	1	8.33	5	50.00	0	0	6	22.22
	− 99	0	0	0	0	0	0	0	0
Enrichment materials	0	0	0	0	0	0	0		
	1	0	0	0	0	0	0	0	0
	2	12	100.00	10	100.00	5	100.00	27	100.00
	− 99	0	0	0	0	0	0	0	0
Shoe hygiene	0	3	25.00	2	20.00	2	40.00	7	25.93
	1	2	16.67	0	0	0	0	2	7.41
	2	7	58.33	8	80.00	3	60.00	18	66.67
	− 99	0	0	0	0	0	0	0	0
Anteroom	0	0	0	0	0	0	0	0	0
	1	1	8.33	1	10.00	0	0	2	7.41
	2	11	91.67	9	90.00	5	100.00	25	92.59
	− 99	0	0	0	0	0	0	0	0
Rodent control	0	0	0	0	0	1	20.00	1	3.70
	1	5	41.67	3	30.00	3	60.00	11	40.74
	2	7	58.33	7	70.00	1	20.00	15	55.56
	− 99	0	0	0	0	0	0	0	0

Scores: 0 = “no biosecurity measures have been implemented, low biosecurity”, 1 = “some biosecurity measures have been implemented, moderate biosecurity”, 2 = “all necessary biosecurity measures have been implemented, high biosecurity”, − 88 = not applicable, − 99 = missing data

**Table 4:** Implementation of biosecurity measures at different farm sites of fattening pig farms

Biosecurity subset	Score	At main site		Next to main site		At separate site		At leased farm site		All	
		n	%	n	%	n	%	n	%	n	%
*Fattening pig farms (n = 38)*											
Perimeter fence	0	10	58.82	2	66.67	9	75.00	5	83.33	26	68.42
	1	4	23.53	0	0	0	0	1	16.67	5	13.16
	2	3	17.65	1	33.33	3	25.00	0	0	7	18.42
	− 99	0	0	0	0	0	0	0	0	0	0
Building structure	0	0	0	0	0	0	0	0	0	0	0
	1	1	5.88	0	0	0	0	2	33.33	3	7.89
	2	16	94.12	3	100.00	12	100.00	4	66.67	35	92.11
	− 99	0	0	0	0	0	0	0	0	0	0
Pathways/vehicle hygiene	0	2	11.76	0	0	0	0	1	16.67	3	7.89
	1	6	35.29	2	66.67	7	58.33	4	66.67	19	50.00
	2	9	52.94	1	33.33	5	41.67	1	16.67	16	42.11
	− 99	0	0	0	0	0	0	0	0	0	0
Loading ramps	0	5	29.41	0	0	1	8.33	4	66.67	10	26.32
	1	3	17.65	1	33.33	0	0	0	0	4	10.53
	2	8	47.06	2	66.67	10	83.33	2	33.33	22	57.89
	− 99	1	5.88	0	0	1	8.33	0	0	2	5.26
Feed silos	0	5	29.41	1	33.33	2	16.67	3	50.00	11	28.95
	1	9	52.94	1	33.33	6	50.00	3	50.00	19	50.00
	2	3	17.65	1	33.33	4	33.33	0	0	8	21.05
	− 99	0	0	0	0	0	0	0	0	0	0
Enrichment materials	0	3	17.65	0	0	1	8.33	0	0	4	10.53
	1	0	0	0	0	0	0	0	0	0	0
	2	14	82.35	3	100.00	11	91.67	6	100.00	34	89.47
	− 99	0	0	0	0	0	0	0	0	0	0
Shoe hygiene	0	5	29.41	0	0	0	0	1	16.67	6	15.79
	1	8	47.06	0	0	0	0	3	50.00	11	28.95
	2	4	23.53	3	100.00	12	100.00	2	33.33	21	55.26
	− 99	0	0	0	0	0	0	0	0	0	0
Anteroom	0	0	0	1	33.33	1	8.33	0	0	2	5.26
	1	5	29.41	1	33.33	3	25.00	6	100.00	15	39.47
	2	12	70.59	1	33.33	8	66.67	0	0	21	55.26
	− 99	0	0	0	0	0	0	0	0	0	0
Rodent control	0	2	11.76	0	0	1	8.33	0	0	3	7.89
	1	3	17.65	0	0	5	41.67	3	50.00	11	28.95
	2	12	70.59	3	100.00	6	50.00	3	50.00	24	63.16
	− 99	0	0	0	0	0	0	0	0	0	0

Scores: 0 = “no biosecurity measures have been implemented, low biosecurity”, 1 = “some biosecurity measures have been implemented, moderate biosecurity”, 2 = “all necessary biosecurity measures have been implemented, high biosecurity”, − 88 = not applicable, − 99 = missing data

**Table 5:** Implementation of biosecurity measures on outdoor pig farms

Biosecurity subset	Score	n	%
*Outdoor pig farms (all sites outdoors, n = 16)*			
Perimeter fence	0	15	93.75
	1	0	0
	2	1	6.25
	− 99	0	0
Building structure and pig pastures	0	3	18.75
	1	13	81.25
	2	0	0
	− 99	0	0
Pathways/vehicle hygiene	0	7	43.75
	1	8	50.00
	2	1	6.25
	− 99	0	0
Loading ramps	0	3	18.75
	1	10	62.50
	2	3	18.75
	− 99	0	0
Feed silos	0	3	18.75
	1	3	18.75
	2	0	0
	− 88	10	62.50
	− 99	0	0
Enrichment materials	0	4	25.00
	1	0	0
	2	12	75.00
	− 99	0	0
Shoe hygiene	0	13	81.25
	1	2	12.50
	2	1	6.25
	− 99	0	0
Anteroom	0	6	37.50
	1	5	31.25
	2	5	31.25
	− 99	0	0
Rodent control	0	5	31.25
	1	3	18.75
	2	7	43.75
	− 99	1	6.25

Scores: 0 = “no biosecurity measures have been implemented, low biosecurity”, 1 = “some biosecurity measures have been implemented, moderate biosecurity”, 2 = “all necessary biosecurity measures have been implemented, high biosecurity”, − 88 = not applicable, − 99 = missing data

**Table 6:** Comparison of self-evaluations by the farmers to the results of the biosecurity check

Production type	Biosecurity subset	Self-evaluation compared to checklist evaluation							
		Missing values		Higher than checklist		Equal to checklist		Lower than checklist	
		N	%	N	%	N	%	N	%
Farrow-to-finish farms	Fence	14	29.79	5	10.64	25	53.19	3	6.38
	Building structure and pig pastures	42	89.36	2	4.26	3	6.38	0	0
	Pathways/vehicle hygiene	24	51.06	0	0	13	27.66	10	21.28
	Loading ramps	28	59.57	6	12.77	10	21.28	3	6.38
	Feed silos	34	72.34	1	2.13	12	25.53	0	0
	Storage of enrichment materials	35	74.47	0	0	12	25.53	0	0
	Shoe hygiene	22	46.81	5	10.64	15	31.91	5	10.64
	Anteroom	8	17.02	6	12.77	28	59.57	5	10.64
	Rodent control	35	74.47	2	4.26	5	10.64	5	10.64
Piglet-producing farms	Fence	8	29.63	2	7.41	15	55.56	2	7.41
	Building structure and pig pastures	27	100.00	0	0	0	0	0	0
	Pathways/vehicle hygiene	9	33.33	4	14.81	6	22.22	8	29.63
	Loading ramps	22	81.48	1	3.70	3	11.11	1	3.70
	Feed silos	19	70.37	0	0	8	29.63	0	0
	Storage of enrichment materials	23	85.19	0	0	0	0	4	14.81
	Shoe hygiene	14	51.85	1	3.70	8	29.63	4	14.81
	Anteroom	3	11.11	2	7.41	20	74.07	2	7.41
	Rodent control	22	81.48	0	0	4	14.81	1	3.70
Fattening pig farms	Fence	8	21.05	4	10.53	24	63.16	2	5.26
	Building structure and pig pastures	21	55.26	3	7.89	10	26.32	4	10.53
	Pathways/vehicle hygiene	23	60.53	2	5.26	3	7.89	10	26.32
	Loading ramps	28	73.68	5	13.16	5	13.16	0	0
	Feed silos	29	76.32	0	0	8	21.05	1	2.63
	Storage of enrichment materials	32	84.21	1	2.63	3	7.89	2	5.26
	Shoe hygiene	14	36.84	7	18.42	13	34.21	4	10.53
	Anteroom	6	15.79	8	21.05	18	47.37	6	15.79
	Rodent control	24	63.16	1	2.63	13	34.21	0	0
Outdoor farms	Fence	8	50.00	7	43.75	1	6.25	0	0
	Building structure and pig pastures	4	25.00	7	43.75	5	31.25	0	0
	Pathways/vehicle hygiene	10	62.50	3	18.75	0	0	3	18.75
	Loading ramps	14	87.50	2	12.50	0	0	0	0
	Feed silos	16	100.00	0	0	0	0	0	0
	Storage of enrichment materials	5	31.25	0	0	9	56.25	2	12.50
	Shoe hygiene	6	37.50	4	25.00	6	37.50	0	0
	Anteroom	3	18.75	4	25.00	8	50.00	1	6.25
	Rodent control	11	68.75	1	6.25	3	18.75	1	6.25

There were also concerns about the interpretation of regulations concerning fences: whether the outer walls of barn buildings counted as sufficient barriers, which structures should be fenced, and which materials should be used for the fences.

### Building structure of pig barns and fences of pastures

Fattening pig and outdoor farmers evaluated the building structure of their pig barns and pig pastures more often than piglet producers and farrow-to-finish farmers (Table 6). Fattening pig farmers considered closed stables to be a great advantage in terms of biosecurity in comparison to outdoor pig farms. The results of the biosecurity check support the farmers’ mostly positive evaluations regarding the structure of their pig barns (score 2: ftf: 74.5% n = 35, pi: 85.2% n = 23, fa: 92.1% n = 35). In contrast, seven outdoor pig farmers (43.8% of farm sites) evaluated the biosecurity provided by the double fence around the pig pasture better than the first author did in the biosecurity check (farmers’ evaluations: Score 2: 37.5% n = 6, score 1: 37.5% n = 6, score 0: 0%, no mention: 25.0% n = 4). A double fence around the pig pastures serves a purpose similar to barn walls, keeping people and wild animals apart from the domestic pigs. Most outdoor farmers were confident that the double fences around their pig pastures were sufficient because they considered the possibility of carrion eating birds or rodents introducing ASFV as less important than the authors. The risk that the authorities could prohibit outdoor pig farming completely, in case wild boar in the area were infected with ASF, was perceived as a greater threat for the existence of the farm.

“However, of course we also have to make sure that we get away from free-range status faster than ASF is here. That is actually our most urgent goal now, because the district office has already told us in no uncertain terms that if there is ASF and we are free-range farmers, then they would withdraw our permit for free-range farming.” (Interview-nr. 25, of)

### Farm grounds and vehicle hygiene

The farmers were very aware of the possible risk of introducing disease agents due to crossing paths on farm grounds, especially at older farm sites and in piglet producing systems (Table 6).

“ (…) the risk is actually that we could carry something over because we (…) drive through the forest with the tractor beforehand and then drive into the pig pasture. So I say that I have to live with that risk, there’s nothing I can do about it anyway.” (Interview-nr. 6, of)

According to the results of the biosecurity check, most farmers implemented measures to prevent their animals from crossing paths with vehicles or wild boar (Tables 2, 3, 4, 5). However, at many sites external vehicles had to cross farm grounds to reach feed silos or loading areas sites (ftf: 97.9% n = 46, pi: 92.6% n = 25, fa: 100% n = 38, of: 95.7% n = 15), which the farmers also considered to be a risk for transmission (Table 6).

### Loading areas

Although the farmers perceived buying new pigs or contact with animal transport vehicles as a risk for the introduction of ASFV, they did not mention biosecurity measures in the loading areas as often as other biosecurity measures (Table 6). However, it was very important to them that the drivers of the animal transport vehicles did not enter the barns from the loading ramp. According to the results of the biosecurity check, more than half of the farm sites with indoor housing systems had an adequately secured loading area for pigs (score = 2) (ftf: 57.5% n = 27, pi: 70.4% n = 19, fa: 57.9% n = 22, of: 18.8% n = 3). However, fences around loading ramps were missing in approximately a quarter of the farm sites (ftf: 19.2% n = 9, pi: 25.9% n = 7, fa: 26.3% n = 10, of: 0%). The farmers considered the fencing of loading ramps as an unnecessary nuisance, especially the required height of 1.5 m. Among outdoor pig farms, most farm sites did not have a separate loading ramp. Pig loading and transportation on outdoor farms was mainly performed by the farmers themselves.

### Storage of feed and enrichment materials

Feed and enrichment materials such as green forage, hay and straw were used more in outdoor pig farms but were also mentioned by some farmers with farrow-to-finish farms (Table 6). They perceived the risk of introducing ASFV with contaminated green forage from their own fields or the nearby area as lower than the risk of buying feed from areas further away. Most farmers stressed the importance of storing feed and enrichment materials out of the reach of wild boar and evaluated their storage positively (score 2: ftf: 21.3% n = 10, pi: 0%, fa: 7.9% n = 3, of: 43.8% n = 7), which corresponds with the results of the biosecurity check (Table 6). Accordingly, the feed at farm sites with indoor housing systems was always stored in closed feed silos that were usually fenced (ftf: 74.5% n = 35, pi: 85.2% n = 23, fa: 71.1% n = 27). However, the silos were rarely accessible from the inside area, although this might be necessary in case of technical difficulties (ftf: 17.0% n = 8, pi: 22.2% n = 6, fa: 21.1% n = 8). Many farmers perceived the legal requirement to fence these silos as unnecessary and impractical.

“We have fenced in the feed silos, for example. There I do not see the point, for example, because we don’t take feed from there. These are measures that we have actually only done for the auditors.” (interview nr. 79, fa)

### Shoe hygiene

In general, the farmers showed a great awareness of the risk of carrying infectious materials in their shoes and mentioned shoe hygiene as one of the key factors in the effective prevention of the introduction of ASFV (Table 6).

“With floor mats, disinfection mats, because I guess that a lot is transmitted through shoes and so on. Maybe pay a bit more attention to clothing hygiene, so that you don’t necessarily run through the pigpens with outside shoes to take a quick look at something or something like that” (Interview-nr. 18, ftf)

Farmers were often critical of their own shoe hygiene measures (score 2: ftf: 19.2% n = 9, pi: 18.5% n = 5, fa: 39.5% n = 15, of: 12.5% n = 2). Even though or even because the pig farmers considered shoe hygiene a rather cheap measure that could be implemented quickly, effective shoe hygiene was often not yet part of the daily routine.

“I always calculate with probabilities. I sometimes find all this disinfecting a bit exaggerated, because you always have to check whether it is truly necessary. Of course, if it comes, then it has probably helped, but since the virus is not yet widespread in Germany, we are a bit more relaxed, I would say.” (Interview-nr. 47, ftf)

In addition, the farm layout was perceived as a major obstacle to effective shoe hygiene, especially at farm sites with multiple buildings and at outdoor sites. An efficient layout of the farm site, the fence or lack of and the management of shoe hygiene all help to prevent an introduction of disease agents into the farm with the shoes. The results of a combined analysis of these factors indicate that caretakers often have to cross farm grounds during work (farm sites: ftf: 70.2% n = 33, pi: 59.3% n = 16, fa: 55.3% n = 21, of: 100% n = 16) and that a fence is missing at more than a fifth of these farm sites (ftf: 21.3% n = 10, pi: 29.6% n = 8, fa: 31.6% n = 12, of: 93.8% n = 15). Moreover, at some of these farm sites, no effective hygiene measures for the shoes were provided (score = 0: ftf: 8.5% n = 4, pi: 22.2% n = 6, fa: 13.2% n = 2, of: 81.3% n = 13) or shoes were disinfected without prior cleaning (score = 1: ftf: 8.5% n = 4, pi: 0% n = 0, fa: 21.1% n = 8, of: 12.5% n = 2).

### Anteroom

The farmers mentioned the implementation of an anteroom and the use of farm-specific clothes for over 80% of the farm sites (Table 6) mostly positively (score 2: ftf: 57.5% n = 27, pi: 81.5% n = 22, fa: 50.0% n = 19, of: 37.5% n = 6). For many of the farmers, changing into farm-specific clothes and shoes when entering the pig barns was a matter of course. The results of the biosecurity check support this evaluation, as most of the farm sites provided clean farm-specific clothing and a sufficiently equipped anteroom (see Scoring System) (score 2: ftf: 68.1% n = 32, pi: 92.6% n = 25, fa: 55.3% n = 21, of: 31.3% n = 5). Farmers with piglet production also stressed the importance of structuring the anteroom—providing a clear delimitation of an inside (farm-specific) and outside (street clothes) area—and were, according to results of the biosecurity check, the only ones who had usually implemented it (ftf: 51.0% n = 24, pi: 29.6% n = 8, fa: 73.7% n = 28, of: 62.5% n = 10). Farmers with farrow-to-finish and fattening pig farms said that they were unable to do so at their fattening sites due to a lack of space in the stables. However, in the biosecurity check, the implementation of anterooms did not differ much between the different farm sites (Tables 2, 3, 4, 5).

“We would have the structure for that. We just have to do it. The rooms are there. (…) However, at the moment, when there is nothing, you’re just too lazy.” (Interview-nr. 71, fa)

The outdoor pig farmers considered the delimitation inside the anteroom a futile effort at their farms since on most outdoor farms (93.8%), the caretakers had to cross the outside area between the anteroom and the pig pastures. At six farm sites (37.5%), no farm-specific clothing was available, and the animal caretakers wore their regular work clothes.

### Rodent control

A possible introduction of infectious agents with rodents can be prevented through a regularly scheduled control system. Approximately a quarter of the farmers mentioned rodents, especially rats, as a possible risk for the introduction of animal diseases, such as ASF (Table 6). To comply with legal regulations, more than half of the farm sites with indoor housing had rodent control at regular time intervals (score 2: ftf: 72.3% n = 34, pi: 55.6% n = 15, fa: 63.2% n = 24, of: 43.8% n = 7). Within multiple-site systems, the implementation of rodent control was consistent among all farm sites. However, rodents were often perceived as an uncontrollable risk for the introduction of animal diseases, and some farmers mentioned former cases in which they suspected rats had carried classical swine fever from one farm site to the next.

### Overall biosecurity evaluation by farmers

Approximately half of the farmers were confident that they had implemented sufficient measures overall to prevent the introduction of ASFV into their farms (ftf: 53.6% n = 15, pi: 52.9% n = 9, fa: 40.9% n = 9, of: 50.0% n = 7). A fifth to half of the farmers still saw need for improvement (ftf: 32.1% n = 9, pi: 41.2% n = 7, fa: 50.0% n = 11, of: 21.4% n = 3). Some interviewees were not content with their biosecurity and considered their pigs insufficiently protected against ASF (ftf: 10.7% n = 3, pi: 5.1% n = 1, fa: 9.1%, n = 2, of: 28.6% n = 4).

## Discussion

This study noted deficits in specific biosecurity subsets on pig farms in the federal state of Lower Saxony in Germany and provides insight into the multitude of factors influencing farmers’ decisions for or against implementing measures protective against ASF on their farms. It was based on a mixed-method approach [46, 47] and combined the quantitative assessment of on-farm biosecurity with a qualitative approach to understanding farmers’ decision-making concerning the implementation of biosecurity measures for ASF.

### Methodology

The qualitative interview approach allowed an exploration of the complexity of factors influencing farmers in their decisions concerning disease risk management [48]. The quality of the interviews depended very much on the farmers’ openness and willingness to participate, which might have limited this study due to selection bias towards farmers who were more aware of the importance of biosecurity. In addition, due to the open structure of the interviews (i.e., no standardised, concrete biosecurity checklist was gone through), the amount and specificity of farmers’ comments about their biosecurity measures varied strongly between the interviews, and it was not possible to draw decisive conclusions from missing mentions. However, it can be assumed from the results that farmers tended to make more mentions of biosecurity measures that were the cause of uncertainties and questions (e.g., fences and shoe hygiene) and spoke less about measures they had implemented and considered a matter of course (e.g., feed storage and hygiene measures at the loading ramp). This was also observed by Casal et al. in their research on pig farms in Spain, where pig farmers mostly mentioned biosecurity measures they had not yet implemented on their farms [49]. Thus, this study complements recent studies on pig farmers’ attitudes and motivational goals in the context of disease prevention.

The biosecurity check in this study did not differentiate farms by size because it aimed to point out biosecurity deficits based on epidemiological considerations concerning the risk for an introduction of ASF rather than on the German legal requirements for biosecurity measures. Therefore, the identified biosecurity deficits in this study do not necessarily imply that the farmers did not adhere to legal regulations. From an epidemiological point of view, the differentiation of legal biosecurity requirements for farms of different sizes in the Pig Husbandry Hygiene Ordinance (*Schweinehaltungshygieneverordnung* [31]) cannot be justified. It is even possible that the lack of legal requirements for smaller pig farms impedes farmers in implementing measures that would be necessary from an epidemiological point of view. Smallholder pig farms are considered a special challenge regarding the implementation of biosecurity measures [50] and have been described to have less awareness of animal disease risks [39, 51]. Further research is needed to estimate the risk these small farms pose in Germany.

The farm site was chosen as the statistical unit because differently structured farm sites were expected to have different prerequisites. However, the results show that the farmer himself also has a major effect on how biosecurity measures are implemented on the farm, especially measures that require little structural change and rely primarily on management.

### Results

In the interviews, most farmers were confident that their own biosecurity measures would be sufficient to protect their animals from ASF. In accordance with the farmers’ perception and previous research [32, 33], the results of the biosecurity check indicate a high level of biosecurity and hygiene routines in most of the participating pig farms. For all production types, the results of the biosecurity check usually correlated with the farmers’ perception of relevant biosecurity subsets. Most deficits were found and discussed in the subsets fence, shoe hygiene and the structure and management of the anteroom (scores varied between the groups). In addition, farmers with outdoor pig farms often discussed the fencing of their pig pastures and the storage of green forage.

According to the results of the biosecurity check, farrow-to-finish and piglet-producing farms provided a slightly higher level of biosecurity than fattening and outdoor pig farms. This could be because farmers with piglet production have a higher interest in preventing ASF introduction than farmers with fattening pigs. According to the pig farmers, piglet-producing farms have to expect much higher costs for rebuilding after an outbreak of ASF than fattening pig farms. In Germany, farmers receive financial aid from the Animal Disease Fund for replacing culled animals, cleaning and disinfection measures in case of an outbreak of ASF on their farm as long as their farm biosecurity meets the legal biosecurity requirements [31, 52]. In addition, many farmers have taken out private animal health insurance that replaces the subsequent financial losses in the non-productive months until the farmer is allowed to buy new animals and rebuild the herd, depending on the individual insurance policy. However, many farmers with piglet production expressed concerns that the financial compensation would not be enough to cover the necessary time and costs of rebuilding a productive herd of sows. In contrast, farmers with fattening pigs were less concerned and more confident that they would receive sufficient compensation.

Many farmers named legal requirements as their main motivation to implement biosecurity measures. This indicates that the German legal system encourages the implementation of biosecurity measures. However, according to the results of the biosecurity check, many farm sites were not adequately fenced, a deficit that could be attributed to the fact that a fence is legally not required for smaller pig farms (fewer than 700 fattening pigs, fewer than 100 (farrow-to-finish)/150 (farrow-to-weaning) sows) [31]. As a fence is an important protective measure against the introduction of ASF—independent of the herd size [28, 53]—the fact that so many farm sites were not fenced is alarming. Many of the pig farmers criticised the legal differentiation by farm size and considered smaller pig farms to be a high risk for the introduction of animal diseases.

Lack of knowledge is known to hinder the implementation of protective measures [38, 54]. In our study, the pig farmers were generally well informed about ASF and the necessary biosecurity measures [35]. Moreover, their evaluation of the implemented biosecurity measures on their farms generally corresponded to the findings of the first author. This shows a great awareness and realistic assessment of their own biosecurity. The few disparities in the evaluation of a biosecurity measure could be attributed to a conflict in the perceived importance of the measure (i.e., structuring of the anteroom) or a different interpretation of its effectiveness (e.g., disinfectant for shoes). Some farmers regarded disinfectant mats for their shoes without prior cleaning as an effective preventive measure. However, disinfectants that are effective against ASFV require a minimum exposure time of 10 min [55], and the Committee of the German Veterinary Medicine Society (DVG, *Deutsche Veterinärmedizinische Gesellschaft e.V.*) recommends soaking shoes for at least 30 min in an approved disinfectant ([56] listed under 7b) after prior cleaning, which is unrealistic for the use of disinfectant mats during daily work routines. In some cases, insecurities about the contagiousness of the ASFV and possible ways of transmission led to a fatalistic attitude among farmers. For example, some farmers, outdoor pig farmers and farmers with indoor housing systems, expressed concerns about rats and carrion-eating birds, which they perceived as uncontrollable risk factors despite rodent control systems, introducing infectious materials. Similar to their risk perception concerning the classical swine fever virus (CSFV), the farmers sometimes overestimated the contagiosity and risks of the ASFV. However, epidemiological research in farms recently affected by ASF in Estonia, Poland and other countries has not yet been able to confirm or dismiss scavengers as possible risk factors [13, 21]. Research on carcasses of wild boar in Germany has suggested that scavengers such as foxes and ravens can carry smaller pieces of meat further than a few metres away from the carcasses [24]. Even though the risk of carrion-eating birds dropping small pieces of infectious materials into a pig pasture cannot be ruled out if wild boar in the immediate proximity of the pasture are infected, the likelihood of such an entry way is much lower than the introduction by human activities [25]. Therefore, farmers’ perception of rats or birds as a high risk seems exaggerated. However, this exaggeration could also be a way of trying to lighten the responsibility to take appropriate preventive measures by attributing the threat of ASF to a risk factor that cannot be influenced. In general, however, the farmers showed a realistic perception of possible risk factors. Despite farmers’ awareness of and knowledge about ASF [35], the epidemiology of ASF and effective disease surveillance methods should remain part of the current knowledge transfer efforts to prevent the development of misunderstandings and uncertainties.

The farmers used a wide spectrum of information sources, and many regarded it as their responsibility to stay well informed about ASF [35]. At the same time, they sometimes felt like advice and information was forced on them. This can cause a rejectionist attitude and impede knowledge transfer [57, 58]. Therefore, advice about biosecurity and ASF should remain a voluntary and easily accessible offer for farmers. In addition, communication in the context of disease risk management should focus on putting the existing knowledge into practice. Previous research on farmers’ attitudes towards information sources identified farm veterinarians as one of the most important sources of information and advice for farmers due to a high level of trust and mutual respect [37]. Therefore, veterinarians and other on-farm advisors could contribute to training in communication aimed at collaboration and a “farmer-centred” approach and thus support farmers even better in protecting their farms from ASF [59].

Regarding the results of the biosecurity subsets in more detail, the farmers named the layout of the farm site and resulting costs as major obstacles to the implementation of a perimeter fence. However, according to the results of the biosecurity check, the implementation of fences was usually consistent within multisite systems, despite the different layouts of the farm sites. These findings suggest that the implementation of measures that require long-term planning and construction depends more on a farmer’s general attitude and motivation to implement effective preventive measures for diseases such as ASF than on the layout of the farm site. On the other hand, the implementation of shoe hygiene measures differed between different farm sites within multisite systems; they were less often implemented in main and leased farm sites than in separately standing farm buildings. The farmers perceived changing shoes as something that could easily and cheaply be implemented but was an unnecessary complication of their work routines as long as the risk of being affected by ASF remained low. The fact that cheap biosecurity measures were also postponed suggests that financial costs also have a lesser influence on farmers’ motivation to change work routines than their general attitude towards biosecurity. This also underlines the importance and likely success of a more farmer-centred approach in disease prevention. Taking farmers’ expertise and their individual circumstances into account could increase farmers’ willingness to implement the necessary biosecurity measures. In addition, a communication platform for farmers could help increase farmers’ motivation and give them new ideas for individual biosecurity solutions. For example, farmers could post pictures or videos online and exchange ideas via an online communication platform. The results from the interviews showed how differently the farmers judged and overcame obstacles to biosecurity implementation.

## Conclusion

The aim of this study was to evaluate the level of biosecurity on pig farms in Lower Saxony with special regards to preventing the introduction of ASFV. In addition, it aimed to elicit factors influencing pig farmers in their decisions concerning the implementation of biosecurity measures. The biosecurity check showed that on most farms, a high level of biosecurity had been implemented. Most deficits were found concerning the fences and the delimitation of clean and dirty areas on farm grounds and in the anteroom. Overall, the farmers had a realistic perception of their own biosecurity. They considered the farm layout one of the main barriers to implementing biosecurity measures against ASF. However, the results also suggested that farmers’ attitudes and legal regulations were major influencing factors. Although pig farmers were well informed about ASF and necessary biosecurity measures, up-to-date information and a constant offer of advice are essential for effective disease risk management. Current knowledge transfer needs to build on farmers’ own motivations and expertise and support them in putting existing knowledge into practice.

### Supplementary Information

**Additional file 1.** "Recruitment flow chart".

**Additional file 2.** "Original interview questionnaire in German language", "Interview questionnaire translated into English language" Interview guide in original language (German) and translated into English.

**Additional file 3.** "ASP-Projekt Erhebungsbogen externe Biosicherheit" Checklist for evaluation of external farm biosecurity in German.

**Additional file 4.** "Scoring-System for the Evaluation of the Biosecurity Checklist" Scoring System in English.

## Data Availability

The data were available through university intern cooperation. Therefore, any data transfer to interested persons is not allowed without an additional formal contract. Data are available to qualified researchers who sign a contract with the University of Veterinary Medicine Hannover. This contract will include guarantees to the obligation to maintain data confidentiality in accordance with the provisions of the German data protection law. Currently, there exists no data access committee or another body who could be contacted for the data. However, for this purpose, a committee will be founded. This future committee will consist of the authors as well as members of the University of Veterinary Medicine Hannover. Interested cooperative partners, who are able to sign a contract as described above, may contact: PD Dr. Amely Campe, Department of Biometry, Epidemiology and Information Processing, University of Veterinary Medicine, Hannover, Buenteweg 2, 30,559 Hannover. Email: amely.campe@tiho-hannover.de.

## Abbreviations

ASF: African swine fever
ASFV: African swine fever virus
CSFV: Classical swine fever virus
TSK: Tierseuchenkasse (Animal Disease Fund)
AHL: Animal Health Law
Ftf: Farrow-to-finish farms *(German: Kb – Kombi-Betrieb)*
Pi: Piglet producers *(German: Fe – Ferkelerzeuger)*
Fa: Fattening pig farms *(German: Mb –Mastbetrieb)*
Of: Outdoor farms *(German: Fh – Freilandhaltung)*
