# Supplementary material for: Biosecurity measures for the prevention of African swine fever on German pig farms: comparison of farmers’ own appraisals and external veterinary experts’ evaluations
Source: Porcine Health Manag. 2024 Mar 11;10:14. doi: 10.1186/s40813-024-00365-x (PMC10926670; doi:10.1186/s40813-024-00365-x)

Additional Material related to publication:

Biosecurity measures for the prevention of African swine fever on German pig farms: Comparison of farmers' own appraisals and external veterinary experts' evaluations

Leonie Klein, Ursula Gerdes, Sandra Blome, Amely Campe, Elisabeth grosse Beilage

Recruitment Flow Chart

Recruitment Flow Chart

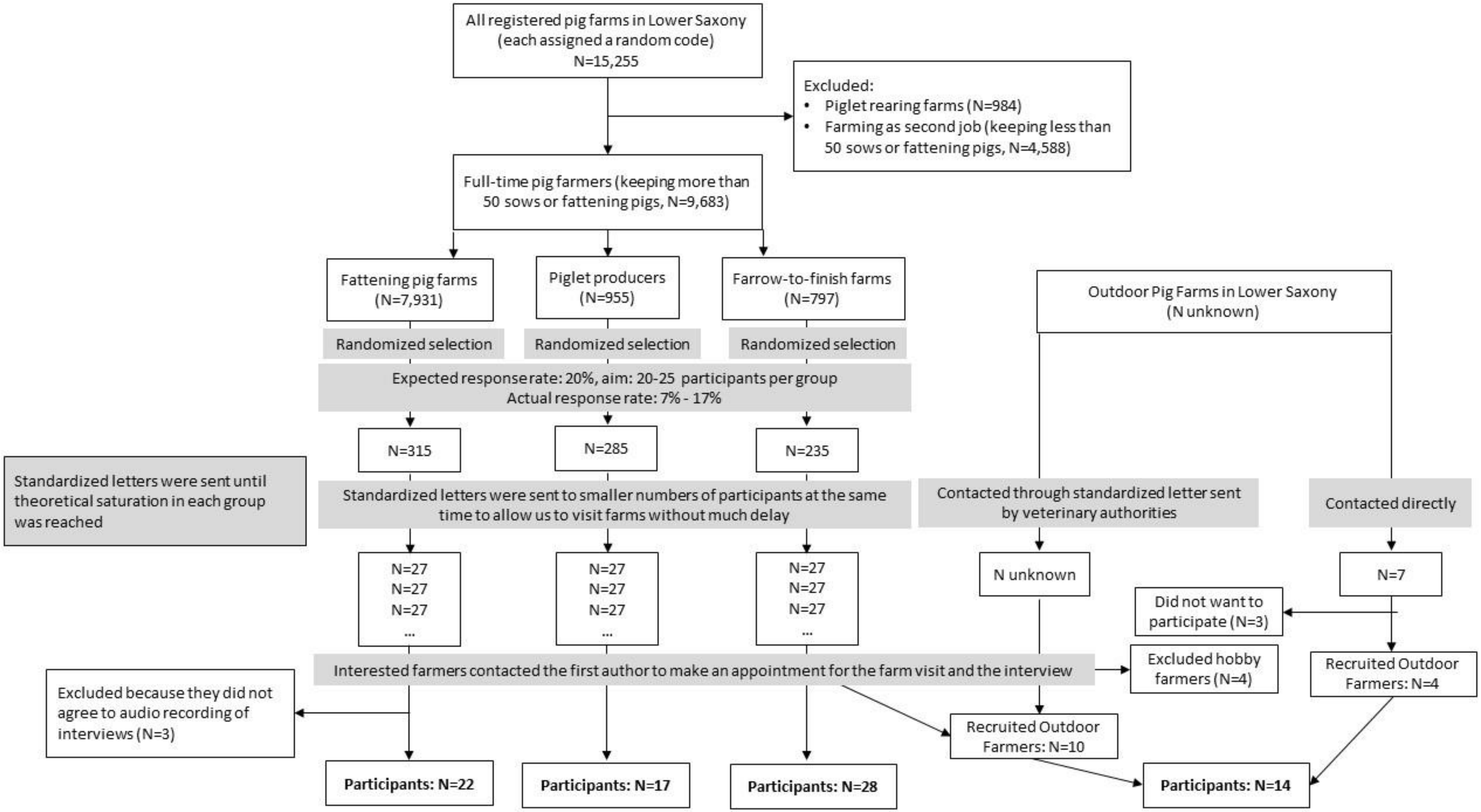

Supplement: Supplementary file 1 — Additional file 1. "Recruitment flow chart". [file 40813_2024_365_MOESM1_ESM.pdf]
