# Supplementary material for: Biosecurity measures for the prevention of African swine fever on German pig farms: comparison of farmers’ own appraisals and external veterinary experts’ evaluations
Source: Porcine Health Manag. 2024 Mar 11;10:14. doi: 10.1186/s40813-024-00365-x (PMC10926670; doi:10.1186/s40813-024-00365-x)
Supplement: Supplementary file 2 — Additional file 2. "Original interview questionnaire in German language", "Interview questionnaire translated into English language" Interview guide in original language (German) and translated into English. [file 40813_2024_365_MOESM2_ESM.pdf]

Additional Material related to publication:

Biosecurity measures for the prevention of African swine fever on German pig farms: Comparison of farmers' own appraisals and external veterinary experts' evaluations

Leonie Klein, Ursula Gerdes, Sandra Blome, Amely Campe, Elisabeth grosse Beilage

Supplement 1: Original interview questionnaire in German language

Supplement 2: Interview questionnaire translated into English language

(Questionnaire used, original German version)

Interview Fragebogen

Leitfaden Betriebsbesuch -> Einleitung

Haben Sie noch Fragen zu dem Projekt oder zu dem Interview?
Wenn Nein -> Können wir starten?

Bedeutung von ASP für den Landwirt

**1. Können Sie mir ein bisschen über Ihren Betrieb erzählen?**	
Wie hat sich Ihr Betrieb entwickelt?<br><br>Wie kam es dazu, dass Sie Schweine halten?<br><br>Wo sehen Sie Ihren Betrieb in 5 Jahren?<br><br>Wie stellen Sie sich die Zukunft Ihres Betriebes vor?	Motivation des Landwirtes<br><br>persönliche Perspektive
Die Afrikanische Schweinepest kommt seit mehr als 10 Jahren wieder auf dem europäischen Festland vor und wird auch in Deutschland immer mehr zum heißen Thema in der Landwirtschaft.	
**2. Welche Bedeutung hat die ASP in der jetzigen Situation für Sie als Schweinehalter?**	
Welche Auswirkungen hätte ein Ausbruch bei Wildschweinen in Niedersachsen auf Ihren Betrieb?<br><br>Wie wäre die Situation für Sie bei einem Ausbruch in einem Hausschweinebestand?<br><br>Was ist für Ihren Betrieb der größtmögliche Schaden durch ASP?	Schwerpunktsetzung durch den Landwirt<br><br>risk perception<br><br>Konsequenzen eines ASP-Ausbruchs<br><br>Biosicherheit

ASP & Biosicherheit

**3. Können Sie sich vorstellen, wie das Virus möglicherweise zu Ihren Schweinen gelangen könnte?**	
Wo sehen Sie für Ihren Betrieb das größte Risiko für einen Eintrag?	Epidemiologie ASPV<br><br>Biosicherheitslücken<br><br>Schwierigkeiten bei der Umsetzung

Überall wird auf die Einhaltung der Biosicherheit hingewiesen, um einen Eintrag der ASP zu verhindern.

**4.**	**Wie schützen Sie Ihren Bestand vor einem Eintrag der ASP?**	
	Welche der Maßnahmen, die Sie mir jetzt genannt haben, sind für Sie denn besonders wichtig?<br><br>Was würden Sie bei einem ASP-Ausbruch verändern/ anders machen?	Glaube an die Effektivität von Biosicherheit<br><br>Welche Biosicherheitsmaßnahmen werden umgesetzt?<br><br>Bewertung

Social ecology, self-efficacy

**5.**	**Welchen Herausforderungen begegnen Sie, wenn es darum geht, Ihren Bestand vor der ASP zu schützen?**	
	Können Sie mir ein Beispiel für etwas nennen, dass sich für Sie gut umsetzen lässt?<br><br>Können Sie mir ein Beispiel nennen, was für Sie schwer umzusetzen ist?<br><br>Haben Sie das Gefühl, dass Ihr Bestand momentan vor einem Eintrag geschützt ist?	Welche Maßnahmen sind schwierig umzusetzen?<br><br>äußere Einflussfaktoren<br><br>Besuchermanagement<br><br>Politik

Informationsquellen

**6.**	**Woher bekommen Sie Ihre Informationen über ASP?**	
	Was interessiert Sie dabei besonders?<br><br>In welchen Situationen sprechen Sie mit anderen Leuten über die ASP?<br><br>Wessen Meinung ist Ihnen dabei besonders wichtig?<br><br>Wie würden Sie Informationen über ASP gerne erhalten?<br><br>Haben Sie Vorschläge, was man verbessern könnte, damit Informationen über ASP besser wahrgenommen werden?	Informationsquellen<br><br>Vertrauenspersonen<br><br>Verbesserungsvorschläge

External investment

**7.**	**Was müsste getan werden, um Sie besser beim Schutz Ihres Betriebs zu unterstützen?**	
	Was müsste getan werden, um Ihren Betrieb besser vor einem ASP-Eintrag zu schützen?<br><br>Wer könnte Ihnen dabei helfen?<br><br>Was müsste getan werden, um Sie vor dem größtmöglichen Schaden durch ASP zu schützen?<br><br>Wer könnte dabei helfen?<br><br>Haben Sie alternative Lösungen, wie es bei einem ASP-Ausbruch trotzdem weitergehen kann?	Internationaler Handel<br><br>Grenzschutz<br><br>Finanzielle Unterstützung<br><br>Informationen
**8.**	**Was hat sich für Sie seit dem ersten ASP-Ausbruch verändert?**	
	Gibt es Fragen in dem Interview, die Sie vor dem Ausbruch der ASP in Brandenburg anders beantwortet hätten?<br><br>*Wenn Ja:* Welche Fragen sind das? Wie hätten Sie geantwortet?	Vergleich mit Antworten vor dem ersten ASP-Ausbruch

Damit sind wir schon am Ende unseres Interviews angekommen.

Abschluss

**9.**	**Möchten Sie noch etwas hinzufügen?**<br><br>**Haben Sie noch etwas anzumerken, was uns beim dem Thema weiterhelfen könnte?**

Vielen Dank für das nette Gespräch.

Interviewregeln:

Die Fragen werden möglichst so gestellt, wie sie im Leitfaden formuliert sind.

Einführende Worte für die Fragen werden (meistens) ad hoc formuliert und sind an vorher vom Landwirt angesprochenen Themen orientiert. Die Einführung für die erste Frage und den Interviewabschluss sind ausformuliert.

Die Reihenfolge der folgenden Fragen folgt dem Gesprächsfluss, sollte aber möglichst am Interviewleitfaden orientiert sein.

Es sollen alle Themen angesprochen werden. Werden Themen vom Interviewten nicht angesprochen, wird ad hoc nachgefragt.

Das Interview sollte nach der Einführung eine Dauer von 30 Minuten nicht überschreiten.

(Questionnaire used, translated English version)

Interview Guide

Interview guide for farm visit -> Introduction

Do you have any questions about the research project or the interview before we start?
If No -> Are you ready for the interview?

Relevance of ASF for the pig farmer

**1. Could you tell me a little bit about your farm?**	
How did the farm develop?<br><br>How did you decide to keep pigs?<br><br>What do you imagine your farm could look like 5 years from now?<br><br>How do you imagine the future of your farm could look like?	Farmer's motivation<br><br>Personal perspectives
African Swine Fever has been present on the European continent again for the past ten years and is developing into an important topic in the agricultural sector in Germany.	
**2. What relevance does ASF currently have for you as a pig farmer?**	
What consequences would an outbreak of ASF among wild boar in Lower Saxony have for your farm?<br><br>What would be the situation if a domestic pig farm was affected?<br><br>What would be the worst case scenario for your farm?	Thematic focus by the farmer<br><br>risk perception<br><br>consequences of an outbreak of ASF<br><br>biosecurity

ASF & Biosecurity

**3. Can you think of ways that your pigs could come in contact with the virus?**	
Where do you see the highest risk for an introduction on your farm?	Epidemiology of ASF virus<br><br>Deficits in biosecurity<br><br>Difficulties in implementing biosecurity measures

There has been a lot of talk about how to prevent the introduction of ASF into pig farms.

**4. How do you protect your farm against an introduction of ASF?**	
Which of the measures you just listed are the most important to you?<br><br>What would you change in case of an outbreak of ASF nearby?	Perceived efficacy of biosecurity measures<br><br>Actual implementation of biosecurity measures<br><br>Evaluation of own biosecurity

Social ecology, self-efficacy

**5. What challenges do you face when protecting your farm against ASF?**	
Could you give me an example of a measure that you find easy to implement?<br><br>Could you give me an example of a measure that you find hard to implement?<br><br>Do you feel like your farm is currently well protected against an introduction of ASF?	Measures that are difficult to implement<br><br>External influences<br><br>Management of visitors<br><br>Politics

Sources of information

**6. From which sources do you receive information about ASF?**	
What information are you especially interested in?<br><br>In what situations do you talk to other people about ASF?<br><br>Whose opinion do you value the most?<br><br>How would you prefer to receive information about ASF?<br><br>Do you have suggestions on how information about ASF could be improved in order to grab people’s attention?	Sources of information<br><br>Important referents<br><br>Suggestions for improving information

External investment

7.	What should be done to better support you in the protection of your farm?	
	What should be done to better protect your farm against an introduction of ASF?<br><br>Who could help you with that?<br><br>What should be done to protect you from the worst-case scenario connected to ASF?<br><br>Who could help you with that?<br><br>Do you have ideas for an alternative future for your farm after a possible outbreak of ASF?	Internationaler Handel<br><br>Grenzschutz<br><br>Finanzielle Unterstützung<br><br>Informationen
8.	What changed for your after the first outbreak of ASF in Germany?	
	Would you have answered any of the questions in the interview differently before?<br><br>If Yes: Which questions? What would you have said?	Comparison to answers before the first outbreak of ASF in Germany

We have reached the end of our interview.

Conclusion

9.	Would you like to add anything?<br>Do you have any comments that would help us further when discussing this topic?

Thank you very much for the interesting interview.

General guidelines for the interview:

Questions are asked as they have been formulated in the questionnaire (as far as possible).

Introductions to the questions are asked ad hoc and are based on what the farmer answered to the previous question. Only the introduction to the first question and the conclusion should be asked as stated in the questionnaire.

The order of the questions follows the questionnaire as much as possible but also considers the natural flow of the conversation.

All topics mentioned in the interview questionnaire should be addressed in the course of the interview. If a topic is not addressed by the interviewee, the interviewer asks ad hoc.

After the introductory question (Question 1), the interview should not last more than 30 minutes.
