# Supplementary material for: Biosecurity measures for the prevention of African swine fever on German pig farms: comparison of farmers’ own appraisals and external veterinary experts’ evaluations
Source: Porcine Health Manag. 2024 Mar 11;10:14. doi: 10.1186/s40813-024-00365-x (PMC10926670; doi:10.1186/s40813-024-00365-x)
Supplement: Supplementary file 3 — Additional file 3. "ASP-Projekt Erhebungsbogen externe Biosicherheit" Checklist for evaluation of external farm biosecurity in German. [file 40813_2024_365_MOESM3_ESM.pdf]

Additional Material related to publication:

Biosecurity measures for the prevention of African swine fever on German pig farms: Comparison of farmers' own appraisals and external veterinary experts' evaluations

Leonie Klein, Ursula Gerdes, Sandra Blome, Amely Campe, Elisabeth grosse Beilage

ASP-Projekt

Erhebungsbogen externe Biosicherheit

ASP-Projekt
Erhebungsbogen externe Biosicherheit

Datum: ___________________

Interne Nummer: _____ Gruppe: _____ Standortnr.: _______

Produktionsstufe: __________ Standortcharakter: _______________________________________

		Ja	Nein	k. A.	Trifft n. zu	Weiß nicht	N. z. beurt.
	**Betriebsgelände**						
**1.**	Trennung in Schwarz- und Weißbereich auch außerhalb des Stalls bzw. Tierbereichs	☐	☐	☐			
**2.**	…ist wildschweinsicher eingezäunt	☐	☐	☐			
**3.**	wildschweinsichere Tore vorhanden	☐	☐	☐			
**4.**	Tore sind über Nacht geschlossen	☐	☐	☐	☐	☐	
	**Hygieneschleuse**						
**5.**	…liegt direkt vor dem Weißbereich.	☐	☐	☐	☐		
**6.**	…ist eindeutig in Schwarz- und Weißbereich getrennt	☐	☐	☐	☐		
**7.**	…ist sauber	☐	☐	☐	☐		
**8.**	betriebseigene Kleidung für Mitarbeiter vorhanden	☐	☐	☐			
**9.**	**saubere** betriebseigene Kleidung für Mitarbeiter vorhanden	☐	☐	☐	☐		
**10.**	betriebseigene Kleidung für Besucher vorhanden	☐	☐	☐			
**11.**	**saubere** betriebseigene Kleidung für Besucher vorhanden	☐	☐	☐	☐		
**12.**	Einwegoveralls vorhanden	☐	☐	☐			
**13.**	betriebseigenes Schuhwerk für Mitarbeiter vorhanden	☐	☐	☐			
**14.**	betriebseigenes Schuhwerk für Besucher vorhanden	☐	☐	☐			
**15.**	Einweg-Überziehschuhe vorhanden	☐	☐	☐			
**16.**	Handwaschbecken vorhanden	☐	☐	☐			

ASP-Projekt
Erhebungsbogen externe Biosicherheit

Datum: ___________________

Interne Nummer: _____ Gruppe: _____ Standortnr.: _______

Produktionsstufe: __________ Standortcharakter: _______________________________________

		Ja	Nein	k. A.	Trifft n. zu	Weiß nicht	N. z. beurt.
**17.**	Handwaschbecken wird offensichtlich regelmäßig benutzt	☐	☐	☐	☐		☐
**18.**	Seife vorhanden	☐	☐	☐			
**19.**	Seife wird offensichtlich regelmäßig benutzt	☐	☐	☐	☐		☐
**20.**	Dusche vorhanden	☐	☐	☐			
**21.**	Dusche wird offensichtlich regelmäßig benutzt	☐	☐	☐	☐		☐
**22.**	Desinfektionsmöglichkeit für Material z.B. Impfstoffe, Medikamente, Werkzeugkoffer vorhanden	☐	☐	☐			
**23.**	Desinfektionsmöglichkeit wird offensichtlich regelmäßig genutzt	☐	☐	☐	☐		☐
**24.**	**Wechsel vom Schwarz- zum Weißbereich**	☐	☐				
**25.**	Die Schuhe werden gewechselt.	☐	☐	☐	☐		
**26.**	für jeden Mitarbeiter an jedem Stalleingang Schuhwerk vorhanden	☐	☐	☐	☐		
**27.**	für Besucher an jedem Stalleingang Schuhwerk vorhanden	☐	☐	☐	☐		
**28.**	Einweg-Stiefelüberzieher vorhanden	☐	☐	☐	☐		
**29.**	Vorrichtung für Reinigung der Schuhe vorhanden	☐	☐	☐			
**30.**	wird offensichtlich regelmäßig benutzt	☐	☐	☐	☐		☐
**31.**	Bei Stiefelreinigern: wird offens. regelmäßig gereinigt	☐	☐	☐	☐		☐
**32.**	Vorrichtung zur Desinfektion der Schuhe vorhanden	☐	☐	☐			
**33.**	wird offensichtlich regelmäßig benutzt	☐	☐	☐	☐		☐
**34.**	Desinfektionsmittel wird offens. regelmäßig ausgetauscht	☐	☐	☐	☐		☐
	**Verkehrswege**						
**35.**	alle Wege sind befestigt, um sie leicht zu reinigen	☐	☐	☐			
**36.**	alle Wege sind sauber	☐	☐	☐	☐		
**37.**	Treibwege der Schweine befinden sich nur im Weißbereich des Betriebsgeländes	☐	☐	☐			
	**Verladerampe**						
**38.**	… ist wildschweinsicher abgesperrt	☐	☐	☐	☐		
**39.**	…ist befestigt, um sie leicht zu reinigen	☐	☐	☐	☐		

ASP-Projekt
Erhebungsbogen externe Biosicherheit

Datum: ___________________

Interne Nummer: _____ Gruppe: _____ Standortnr.: _______

Produktionsstufe: __________ Standortcharakter: _______________________________________

		Ja	Nein	k. A.	Trifft n. zu	Weiß nicht	N. z. beurt.
**40.**	Zurücklaufen der Schweine in den Stall bzw. Tierbereich während der Verladung in den Transporter wird verhindert	☐	☐	☐		☐	
**41.**	Der Tiertransporteur betritt nie den Stall bzw. Tierbereich von der Verladerampe aus.	☐	☐	☐	☐	☐	
	**Futter, Einstreu und Beschäftigungsmaterial**						
**42.**	Futter wird wildschweinsicher gelagert	☐	☐	☐			
**43.**	Futtersilos sind wildschweinsicher eingezäunt.	☐	☐	☐	☐		
**44.**	Mitarbeiter können die Futtersilos ohne Verlassen des Weißbereiches erreichen	☐	☐	☐	☐		
**45.**	Nutzung von **frischem** Grünaufwuchs als Futter, Beschäftigungsmaterial oder Einstreu	☐	☐	☐		☐	
**46.**	**frischer** Grünaufwuchs stammt aus eigenem Anbau	☐	☐	☐	☐	☐	
**47.**	**frischer** Grünaufwuchs wird wildschweinsicher gelagert	☐	☐	☐	☐		
**48.**	Nutzung von **getrocknetem** Grünaufwuchs (Heu oder Stroh) als Futter, Beschäftigungsmaterial oder Einstreu	☐	☐	☐		☐	
**49.**	**getrockneter** Grünaufwuchs stammt aus eigenem Anbau	☐	☐	☐	☐	☐	
**50.**	**getrockneter** Grünaufwuchs wird wildschweinsicher gelagert	☐	☐	☐	☐		
**51.**	Nutzung von Holz oder anderen Materialien aus dem Wald als Beschäftigungsmaterial	☐	☐	☐		☐	
**52.**	Beschäftigungsmaterial wird wildschweinsicher gelagert	☐	☐	☐	☐		
	**Tierbereich/ Stall**						
**53.**	Notausgänge können nur von innen geöffnet werden.	☐	☐	☐	☐		
**54.**	Offene Türen und Fenster sind durch Gitter gesichert.	☐	☐	☐	☐		
**55.**	kein direkter Kontakt zwischen Wild- und Hausschweinen möglich	☐	☐	☐			
**56.**	Vor dem Einflug größerer Vögel wie Krähen geschützt.	☐	☐	☐			
	**Bekämpfung von Ratten**						
**57.**	…wird außerhalb des Stalls bzw. Tierbereichs durchgeführt	☐	☐	☐		☐	
**58.**	Wenn Ja. Wie oft? ____			☐	☐	☐	
**59.**	Von wem? ______________________________________			☐	☐	☐	
**60.**	… wird innerhalb des Stalls bzw. des Tierbereichs durchgeführt	☐	☐	☐		☐	
**61.**	Wenn Ja: Wie oft? ____			☐	☐	☐	
**62.**	Von wem? ______________________________________			☐	☐	☐	

ASP-Projekt

Erhebungsbogen externe Biosicherheit

Datum: ___________________

Interne Nummer: _____ Gruppe: _____ Standortnr.: _______

Produktionsstufe: __________ Standortcharakter: _______________________________________

Frage Nr.	Anmerkung
