# Supplementary material for: Biosecurity measures for the prevention of African swine fever on German pig farms: comparison of farmers’ own appraisals and external veterinary experts’ evaluations
Source: Porcine Health Manag. 2024 Mar 11;10:14. doi: 10.1186/s40813-024-00365-x (PMC10926670; doi:10.1186/s40813-024-00365-x)
Supplement: Supplementary file 4 — Additional file 4. "Scoring-System for the Evaluation of the Biosecurity Checklist" Scoring System in English. [file 40813_2024_365_MOESM4_ESM.pdf]

Additional Material related to publication:

Biosecurity measures for the prevention of African swine fever on German pig farms: Comparison of farmers' own appraisals and external veterinary experts' evaluations

Leonie Klein, Ursula Gerdes, Sandra Blome, Amely Campe, Elisabeth grosse Beilage

Scoring-System for the Evaluation of the Biosecurity Checklist

Translated for the purpose of publication

Biosecurity Area	Evaluation	Score
**1. Fences**		
	Farm grounds are not fenced safely against wild boar.	0
	Farm grounds are fenced safely against wild boar but gates are always open.	1
	Farm grounds are fenced safely against wild boar, including closed gates.	2
**2. Building structure of pig barns and pig pastures**		
	There is the possibility that domestic pigs could come into contact with wild boar. In outdoor farms, the double fencing of animal enclosures is missing.	0
	Buildings have open windows or doors/ are completely open (outdoor farm) but no direct contact between domestic pigs and wild boar is possible.	1
	Completely closed buildings	2
**3. Pathways and Vehicle hygiene**		
	Animals‘ pathways are located outside the clean area.	0
	Farm grounds are not paved or extremely muddy, but animals’ pathways are located only in the clean area.	1
	Farm grounds are paved and clean and animals’ pathways are located only in the clean area.	2
**4. Loading areas**		
	Loading areas are not fenced safely against wild boar.	0
	Loading areas are fenced but there is no clear separation of clean and dirty area.	1
	Loading areas are fenced safely against wild boar and are clearly divided into clean and dirty areas.	2
**5. Feed silos**		
	Feed silos are not fenced and not accessible without leaving the clean area.	0
	Feed silos are fenced but not accessible without leaving the clean area.	1
	Feed silos are fenced and accessible inside the clean area.	2
	Feed is not stored in feed silos.	-88
**6. Storage of feed and bedding materials**		
	Organic enrichment and bedding material is not stored safely out of the reach of wild boar.	0
	Feed is stored safely out of the reach of wild boar, bedding material and organic enrichment material is not in use [1].	2
	Organic enrichment and bedding material is storeed safely out of the reach of wild boar (if from a region free of ASF).	2

**7.**	**Shoe hygiene**	
	Farm grounds (dirty area) are crossed during work routines without additional hygiene measures.	0
	Farm grounds (dirty area) are crossed during work routines with insufficient hygiene measures (desinfection of the shoes, no cleaning, no additional shoes for external personnel).	1
	Farm grounds (dirty area) are crossed during work routines with changing OR cleaning AND desinfection of shoes before and after entering buildings or animal enclosures. OR crossing of farm grounds (dirty area) during work routines is not necessary.	2
**8.**	**Anteroom – Score is calculated as the median of the following three scores:**	
	**Structure of the anteroom**	
	No anteroom available	0
	No clear subdivision of the anteroom into a clean and dirty area or extremely dirty anteroom	1
	Clean anteroom with a clear subdivision into clean and dirty area.	2
	**Change of clothes**	
	No farm specific clothing available	0
	Only farm specific shoes avaiblable or no farm specific clothing for external personnel available	1
	Farm specific shoes and clothing for personnel and external personnel available	2
	**Washing and Showering**	
	No facilities for washing hands or showering available	0
	Insufficient facilities for washing hands or showering available (no water, no soap, dirty towels)	1
	Facilities for washing hands (including soap and clean towels) or showering are available.	2
**9.**	**Rodent control**	
	No rodent control	0
	Rodent control in the outside area if needed without a schedule.	1
	Rodent control in the outside area in established intervals (at least every 3 months)	2

1) Providing the pigs with organic materials for enrichment and bedding (straw, hay, wood or something similar) is a legal requirement according to the “Tierschutznutztierhaltungsverordnung”. However, in how far the pig farmers adhered to such legal requirements was not subject of this research project and therefore not critically evaluated in the scoring system.
